# *mars *and *tousled-like kinase *act in parallel to ensure chromosome fidelity in *Drosophila*

**DOI:** 10.1186/1423-0127-16-51

**Published:** 2009-06-01

**Authors:** Hsing-Hsi Li, Chuen-Sheue Chiang, Hsiao-Yu Huang, Gwo-Jen Liaw

**Affiliations:** 1 Department of Life Sciences and Institute of Genome Sciences, National Yang-Ming University, Taipei, 112 Taiwan, ROC; 2 Centers for Disease Control, Department of Health, Taipei, 115 Taiwan, ROC

## Abstract

**Background:**

High levels of *Hepatoma Up-Regulated Protein *(*HURP*) and *Tousled-Like Kinase *(*TLK*) transcripts are found in hepatocellular carcinoma. *HURP *overexpression induces anchorage-independent growth of 293-T cells and enhances a rough-eye phenotype resulting from *tlk *overexpression in *Drosophila*. In addition, both HURP and Mars, a *Drosophila *HURP sequence homologue, promote polymerization of mitotic spindles. Thus, the genetic interaction of *mars *with *tlk *might be required for accurate chromosome segregation.

**Methods:**

To reveal whether chromosome fidelity was decreased, the frequency of gynandromorphy, an individual with both male and female characteristics, and of non-disjunction were measured in the progeny from parents with reduced *mars *and/or *tlk *activities and analyzed by Student's *t*-test. To show that the genetic interaction between *mars *and *tlk *is epistatic or parallel, a cytological analysis of embryos with either reduced or increased activities of *mars *and/or *tlk *was used to reveal defects in mitotic-spindle morphology and chromosome segregation.

**Results:**

A significant but small fraction of the progeny from parents with reduced *mars *activity showed gynandromorphy and non-disjunction. Results of cytological analysis revealed that the decrease in chromosome fidelity was a result of delayed polymerization of the mitotic spindle, which led to asynchronous chromosome segregation in embryos that had reduced *mars *activity. By removing one copy of *tousled-like kinase *(*tlk*) from flies with reduced *mars *activity, chromosome fidelity was further reduced. This was indicated by an increased in the non-disjunction rate and more severe asynchrony. However, the morphology of the mitotic spindles in the embryos at metaphase where both gene activities were reduced was similar to that in *mars *embryos. Furthermore, *tlk *overexpression did not affect the morphology of the mitotic spindles and the cellular localization of Mars protein.

**Conclusion:**

Chromosome fidelity in progeny from parents with reduced *mars *and/or *tlk *activity was impaired. The results from cytological studies revealed that *mars *and *tlk *function in parallel and that a balance between *mars *activity and *tlk *activity is required for cells to progress through mitosis correctly, thus ensuring chromosome fidelity.

## Background

Genetic instability is a hallmark of cancers and occurs at two levels. First, defects in the DNA repair system increase the mutation rate due to an inability to correct errors resulting from DNA damage or DNA replication. Second, missegregation of chromosomes during mitosis or meiosis leads to aneuploidy or translocations. Chromosome segregation is controlled by groups of proteins acting together in order to coordinate the M-phase progression [1-3]. Mutation of a protein playing a key role during chromosome separation would be expected to lead to cell death. However, when a protein has a loss-of-function mutation and the protein plays a subtle role in mitosis, this may result in viable cells that have chromosome abnormalities and in neoplasia [4].

In the postgenomic era, along with molecular biology tools that have been developed to explore global gene expression profiles, database mining using bioinformatics has revealed key differences in genes expressed under various conditions. One such example is the comparison of genes expressed in human hepatocellular carcinoma (HCC) versus normal liver cells [5,6]. One gene found to be up-regulated at the G2/M transition in human 293T cells from these profiling experiments is *Hepatoma Up-Regulated Protein *(*HURP*), which encodes a protein that contains a guanylate kinase associated protein domain (GKAP) [7]. This domain was initially identified in a protein associated with Postsynaptic density-95 (PSD-95), a member of the membrane-associated guanylate kinase homologue (MAGUK) family. MAGUKs play roles in cytoskeleton signaling and at the synaptic/epithelial cell junctions [8-10]. Overexpression of *HURP *induces anchorage-independent growth of human 293T cells, suggesting that it is involved in tumorigenesis [7].

When cells are ready to divide, they undergo several morphological changes, including centrosome separation, nuclear envelope breakdown and chromosome condensation. These events are coordinately controlled by several highly conserved kinases, such as Cdc2/CyclinB, Polo-like and Aur-A/Ipl1. AUR-A mediates Ran-GTP activity [11], which regulates the stability and activity of HURP by controlling the accessibility of the protein's microtubule-binding domain [1,6]. HURP promotes polymerization of the spindle microtubules near the kinetochore in order to generate sufficient tension across the sister kinetochores [12-14]. In addition, HURP drives the formation of a tubulin (Tub) sheet that wraps around ends of microtubule bundles to strengthen the mitotic spindle [15].

We conducted a gain-of-function screen to search for human genes across 76 selected genes that are up-regulated in HCC. The aim was to identify genes that modify the rough-eye phenotype caused by *tousled-like kinase *(*tlk*) overexpression. *HURP *was found to be one such gene. The *tousled *gene in *Arabidopsis thaliana *encodes a Ser/Thr kinase and is the founding member of the Tlk subfamily [16]. Members in this subfamily are highly conserved from protozoa to mammals [16-20]. Tlks are thought to participate in cancer development; this is based on the facts that human *TLKs *are also up-regulated in a number of cancers, including HCC [21] and that Tlk function during the S phase of the cell cycle in vertebrates [20]. In humans and fly, TLK2 and Tlk, respectively, physically interact with Asf1, a factor required for chromatin assembly [17,22]. Furthermore, in *Caenorhabditis elegans *and *Trypanasoma brucei*, Tlk-1 is a substrate of and mediates activation of the Aur-B kinases [3,19,23], the activity of which promotes amphitelic attachment (each sister kinetochore binds to one of the two spindle microtubules arising from opposite poles) rather than monotelic or syntelic attachment (only one or both sister kinetochores bind to spindle microtubules arising from the same pole) during chromosome congression (the syn-to-amphitelic transition) [24,25].

**Figure 1:**
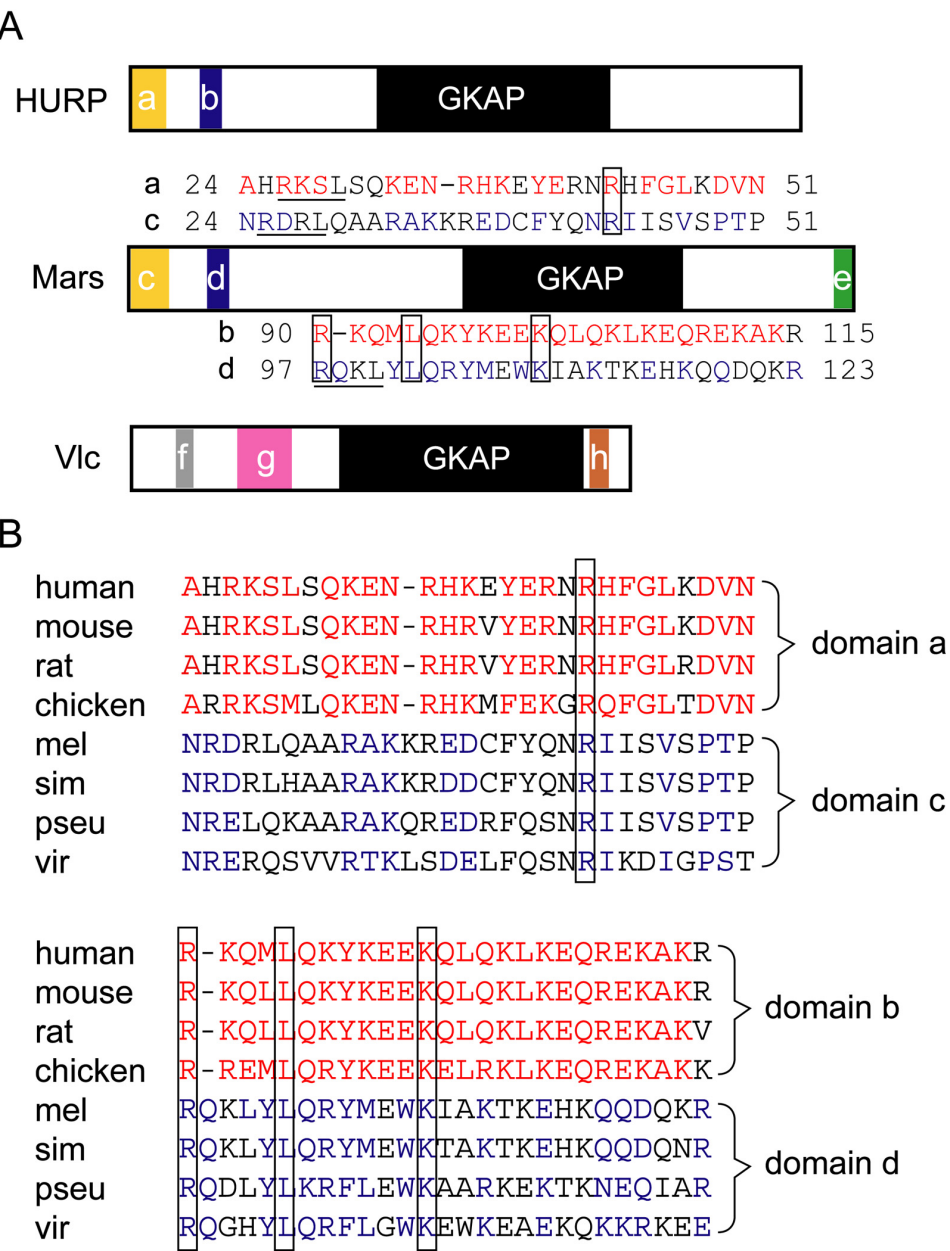
**Similarity between Mars and HURP is higher than that between Vulcan and HURP**. (A) Diagrams show conserved domains in HURP, Mars and Vulcan (Vlc). The sizes of the GKAP domains in HURP, Mars and Vlc are 297 (301–597), 315 (452–766) and 323 (266–588) amino acids, respectively. The amino acid sequences of the HURP orthologues in human, mouse, rat and chicken were aligned to identify conserved domains other than the GKAP domain. Two conserved domains, a and b with similarities of 79% and 92%, were found using the ClustalW program. Similarly, three conserved domains in Mars (c-e) and Vlc (f-h) among *Drosophila *species including *melanogaster, simulans*, *pseudoobscura *and *virilis *were identified. Similarities of these three domains in Mars and Vlc ranged from 57% to 73% and from 90% to 95%, respectively. Paired amino acid sequences of domains a/c and b/d are shown. Conserved amino acids in the HURP or Mars orthologues are indicated by red and blue letters (see sequence alignments in panel B). The highly conserved amino acids among the eight species are boxed. Putative destruction boxes, RXXL, are underlined. (B) The conserved domains in the HURP orthologues from vertebrates (human, mouse, rat and chicken) and in the Mars orthologues from *Drosophila *species, including *melanogaster *(mel), *simulans *(sim), *pseudoobscura *(pseu) and *virilis *(vir) were separately identified using the CustalW program. Sequences of the conserved domains were aligned manually to identify highly conserved amino acid residues (boxed). Conserved amino acids in the HURP and Marsorthologues are indicated by red and blue letters.

In *D. melanogaster*, both *mars *and *vulcan *(*vlc*) encode GKAP-containing proteins [26,27]. Neither Mars nor Vlc shows high sequence homology to HURP except for their GKAP domains. Although the similarity of the GKAP domains between Vlc and HURP is higher than that between Mars and HURP, the relative positions of several other conserved domains, which are found in these three proteins when vertebrates and *Drosophila *are compared, are more similar between Mars and HURP. This contrasts with Vlc and HURP, where there is little similarity in these domains when the situation is compared to Mars and HURP (Fig. 1). Specifically, putative destruction boxes (D boxes, RXXL) are found in the domain a of HURP and in the domains c and d of Mars (Fig. 1) and the latter two are in the region responsible for Mars degradation [28,29]. In addition, *mars *overexpression induces a metaphase arrest and results in abnormal chromosome figures in cells of the eye disc, indicating that Mars is required for accurate chromosome segregation [29]. Furthermore, Mars binds to protein phosphatase 1 to dephosphorylate DrosophilaTransforming acidic coiled-coil protein that stabilizes microtubules [30]. In this study, we characterized the genetic interaction between *mars *and *tlk *and assessed their involvement in chromosome fidelity.

## Methods

### Drosophila strains and fly genetics

Lines w*; *P{EP}drk*^*EP2477*^/*CyO*, *Df*(*2R*)*CX1 wg*^*12 *^*b*^*1 *^*pr*^*1*^/*SM1 *and *w*^*67c23 *^*P{w*^*+MC *^= *lacW}tlk*^*G0113a*, *b*, *c *^were obtained from the Szeged and Bloomington Stock Center. Tan and coworkers has reported that w*; *P{EP}drk*^*EP2477*^/*CyO *is a hypomorph of *mars*, designated as *mars*^*P *^[30]. A *drk *hypomorph, *drk*^*R1*^/*CyO *[31], *y*^*1 *^*w*^*1 *^*sn*^*3*^, *P{w*^*+MC *^= *UAST*-*tlk}#0 *(abbreviated as *UAST*-*tlk*) and *w tlk*^*Δ14*^/*FM7 *[17] were generous gifts from Drs. E. Hafen, H.Y. Sun and F. Karch.

Starting with *w*^*67c23*^* P{w*^*+MC *^= *lacW}tlk*^*G0113a *^in which the other two P-element inserts were segregated by meiotic recombination, a duplication line, *w*^*67c23 *^*tlk*^*27*^, with another copy of *P*{*w*^*+MC *^= *lacW*} inserted at the 5'end of *Rala*, was generated. Using P-element imprecise excision, a deficiency, *w*^*67c23 *^*tlk*^*27-9*^, with a deletion between the 5'end of *Rala *and 4^th ^exon of *tlk-RB*, was obtained. Transgenic fly lines carrying a *P*{*w*^*+MC *^= *UASP*-*tlk*} transgene (abbreviated as *UASP*-*tlk*) were generated by inserting the *tlk *coding region in the GH07910 EST clone into pUASP [32] and then the resulting plasmid DNA was transformed into flies using P-element mediated germ-line transformation [33,34]. To express *tlk *in the germ line, females of a selected *UASP*-*tlk *line were crossed with *GAL4*-*GCN4 *males [35]. The resulting females carrying both transgenes were crossed with *UAST*-*tlk *males for the collection of embryos, which are described here as *GCN4*>*tlk *embryos.

The w*; *P{EP}drk*^*EP2477*^/*CyO *strain obtained from the stock center was homozygous lethal. To eliminate the possible existence of a second-site mutation, meiotic recombination was used [36]. After five rounds of meiotic recombination, some newly generated lines were homozygous viable and P-element insertion was confirmed by PCR.

### Immunoblotting

To determine the level of Mars protein in syncytial blastoderm embryos, a collection of metaphase embryos as described by Su (2000) was made and this collection was used to perform immunoblotting. In brief, embryos from 0 to 1 hour were collected and aged for 1.5 hour. The embryos were dechorionated in bleach, fixed in methanol for 5 minutes and stained with 40 pg/ml of Hoechst 33342 (Sigma/Aldrich, Inc) in 1× PBS. Syncytial blastoderm embryos at metaphase were picked out under an inverted fluorescence microscope (Leica Model DM Illinois, USA) [37]. Protein pools from 30 embryos were separated using 8% SDS polyacrylamide gels. The proteins in the SDS gels were transferred onto PVDF membrane and Mars was detected using anti-Mars antibody (1:5000; generously provided by Dr. S.-S. Fan [38]). After incubation with the secondary antibody, a chemilluminescent assay kit (Western lighting™, Blossom Biotechnologies, Inc, Taiwan) was used to detect the protein [39].

### Immunohistochemical analysis

Embryos were fixed in 37% formaldehyde at room temperature (RT) for 5 min. Eye-antennal discs of late third instar larvae were dissected in 1× PBS and then transferred into 1× PBS containing 4% paraformaldehyde for 30 min. The embryos or eye-antennal discs were washed with 1× PBST (0.3% Triton X-100 in 1× PBS) and incubated with a blocking solution (1% BSA in 1× PBST) prior to incubation with various primary antibodies, anti-phosphohistone H3 (Upstate) (1:200 dilution), α-Tub (Sigma/Aldrich, Inc) (1:200 dilution), γ-Tub (Sigma/Aldrich, Inc) (1:200 dilution), or Mars (1:400 dilution), at either 4°C overnight or at RT for 2 hours. Localization of the proteins was detected by incubating with secondary antibodies conjugated with either Cy3 or FITC (Jakson ImmunoResearch Lab) (1:200 dilution) at 4°C overnight or at RT for 2 hours. The contours of the photoreceptor clusters in the eye discs were stained with Phalloidin-Tetramethylrhodamin B isothiocyanate (Phalloidin-TRITC) (Invitrogen Molecular Probe) (1:80 dilution) for 1 hour. Embryos were incubated with 4 ng/ml of Hoechst 33342 (Sigma/Aldrich, Inc) to stain their chromosomes. The embryos or eye-antennal discs were mounted in a mounting medium (20 mM Tris-HCl pH 8.8, 50% glycerol and 4% n-propyl gallate) and viewed under a Leica confocal microscope (Model TCS-SP2) [39,40].

**Figure 2:**
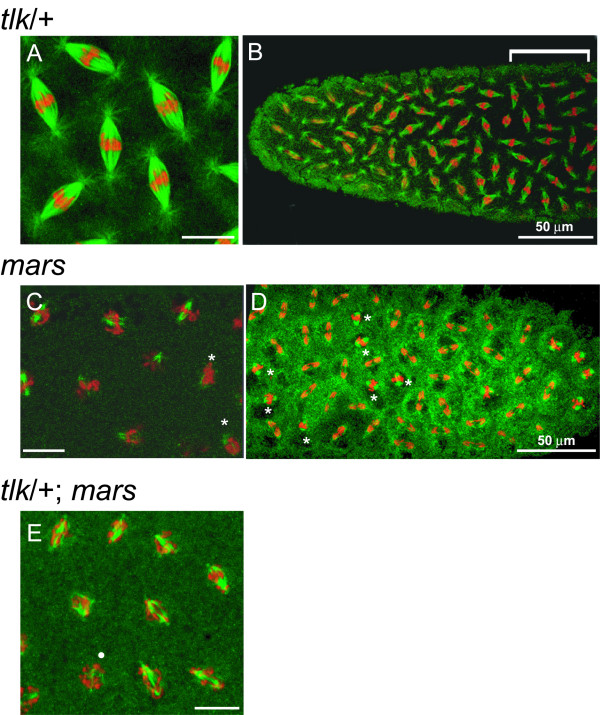
**Asynchronous mitosis occurs in *mars *and *tlk*/*mars *embryos at the non-permissive temperature**. Embryos from *tlk*^*Δ14*^/+ (A and B), *mars*^*P *^(C and D) and *tlk*^*Δ14*^/+; *mars*^*P *^(E) females crossed with *mars*^*P *^males were immunostained as described in the legend of Figure 4. In panel B, chromosomes aligned at spindle midzone before entering anaphase are indicated by a bracket above the embryo. Nuclei with delayed mitotic progression relative to neighboring nuclei at metaphase (C) or anaphase (D) are indicated by asterisks. The chromosome segregation in the *tlk*/*mars *embryos is very asynchronous (E). A white circle indicates a nucleus that is likely at metaphase based on the morphology of chromosomes. The scale bars without specification are 10 μm

To determine the density of the mitotic spindles at metaphase, anaphase or telophase, as represented by the fluorescence intensity of the mitotic spindle, the green channel of the selected images was converted into grayscale and the gray value surrounding the mitotic spindle was adjusted to around 80% using Photoshop. For each mitotic phase, the intensity of one 10 by 10 pixel square of one spindle from each nucleus was measured using Image J . Six nuclei in twelve embryos at nuclear cycles 10 or 11 (as shown in Fig. 2D) were randomly chosen for the measurements, which were used to obtain an average intensity. The average value was then normalized against the background, which was the average value from ten 10 by 10 pixel squares outside of and surrounding the mitotic spindle. This produced values in arbitrary units (au) of fluorescence intensity. The length of the mitotic spindle from centrosome to centrosome of the same nuclei was also measured. The statistical significance of differences in the density and the length of the assessed mitotic spindles were determined using the Student's *t*-test.

## Results

### Mars localizes on the mitotic spindle at metaphase and anaphase

**Figure 3:**
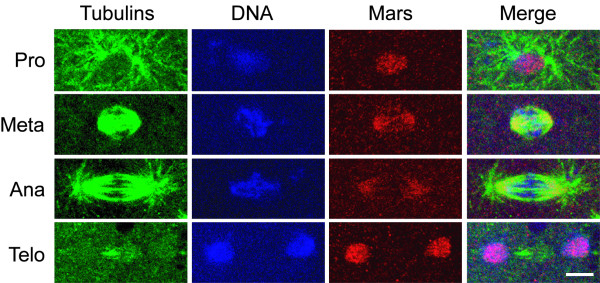
**Mars localizes to mitotic spindles**. *w*^*1118 *^embryos were immunostained with anti-Mars (red), anti-α-Tub (green) and anti-γ-Tub (green) antibodies. The chromosomes were stained by Hoechst 33342 (blue). The different phases of mitosis are indicated on the left. The scale bar is 5 μm.

Immunostaining was performed to reveal the localization of endogenous Mars protein. Throughout the syncytial blastoderm stage, a low level of punctate staining was observed, indicating that a low level of Mars was uniformly present in the cytoplasm of the embryos (data not shown). In addition to this low level and uniform distribution of Mars protein, at the onset of mitosis, when the centrosome is divided into two, a high level of Mars was detected in the nucleus (Fig. 3). During metaphase and anaphase, Mars was predominantly localized on the mitotic spindles, a cellular structure essential for faithful distribution of chromosomes into the two daughter cells [41,42], but not on the centrosomes or the astral microtubules. At telophase, a high level of Mars colocalized with the de-condensing chromosomes. The subcellular localizations of Mars at M phase are consistent with other reports [30,43], indicating that Mars functions on the mitotic spindles.

### Hatching rate of *mars*^*P *^embryos is reduced

Located at 50A13-14, *mars *and *downstream receptor kinase *(*drk*) are transcribed in opposite directions and overlap by 36 bp. The insertion of *P{EP} *at 23 bp upstream of the putative start codon of *mars *in line *mars*^*P*^/*CyO *results in a hypomorphic mutation of *mars *[30]. Since the line obtained from the stock center is homozygous lethal, second-site lethal mutations were segregated out by meiotic recombination. The hatching rate of embryos from parents homozygous for *mars*^*P *^(abbreviated as *mars *embryos hereafter) was 67.0%, while those of embryos from females homozygous for *w*^*1118 *^and heterozygous for *drk*^*R1 *^and *mars*^*P *^were 91.7 or 96.4%, respectively (Table 1). The hatching rate was slightly higher than that reported by Tan et al. (49%, [30]). Furthermore, the viability of the *mars *embryos was temperature-dependent and the hatching rate was further reduced to 39.2% at 28.5°C. These results supported the fact that *mars*^*P *^is a hypomorph and indicated that lethality is due to a shortage of both maternal and zygotic *mars *activity.

**Table 1:** Reduced fertility of *mars *alone and in combination with two *tlk *alleles

Crosses (females × males)	Embryo hatching rate (%)
*w*^*1118 *^× *w*^*1118*^	91.7 ± 4.3
*mars*^*R160 *^× *mars*^*R160*^	90.4 ± 1.4
*w*^*1118*^; *mars*^*P*^/*drk*^*R1 *^× *w*^*1118*^; *mars*^*P*^	96.4 ± 2.6
*w*^*1118*^; *mars*^*P *^× *w*^*1118*^	95.8 ± 2.2 87.0 ± 2.3^a^
*w*^*1118*^; *mars*^*P *^× *w*^*1118*^; *mars*^*P*^	67.0 ± 1.8 39.2 ± 4.3^a^
*w*^*1118*^; *mars*^*P*^/*Df(2R)CX1 *× *w*^*1118*^	0
*w*^*67c23 *^*tlk*^*27-9*^/+ × *w*^*1118*^	97.0 ± 1.1
*w*^*67c23 *^*tlk*^*Δ14*^/+ × *w*^*1118*^	93.3 ± 0.9
*w*^*67c23 *^*tlk*^*27-9*^/+; *mars*^*P*^/*+ *× *w*^*1118*^; *mars*^*P*^	92.8 ± 0.6
*w*^*67c23 *^*tlk*^*Δ14*^/+; *mars*^*P*^/*+ *× *w*^*1118*^; *mars*^*P*^	87.2 ± 2.2 77.5 ± 3.3^a^
*w*^*67c23 *^*tlk*^*27-9*^/+; *mars*^*P *^× *w*^*1118*^; *mars*^*P*^	48.0 ± 1.6
*w*^*67c23 *^*tlk*^*Δ14*^/+; *mars*^*P *^× *w*^*1118*^; *mars*^*P*^	63.8 ± 2.2 42.4 ± 3.8^a^

Age of parents was 3–5 days. At least 600 embryos were scored to determine the hatching rate in each experiment.

a: Fertility was measured at the non-permissive temperature, 28.5 ± 0.5°C.

### *mars* loss-of-function decreases chromosome fidelity

An organism that has a mixture of male and female characteristics is called as gynandromorph. Before sex is determined in a female embryo (X/X), loss of one sex chromosome during mitosis results in X/O cells that eventually lead to a male phenotype at the adult stage [36]. From *mars*^*P *^parents, we found gynandromorphs formed approximately 0.05% of the progeny. Mars plays an important role to stabilize spindle microtubules, and its mutations thus result in the formation of abnormal spindle microtubules [30,38]. Similarly, it has been shown that *nonclaret disjunctional *loss-of-function causes defects in the mitotic spindle, which also results in gynandromorph [44]. Therefore, based on the above results, we examined morphology of mitotic spindles in *mars *embryos.

### *mars* embryos exhibit delayed polymerization of mitotic spindles

**Figure 4:**
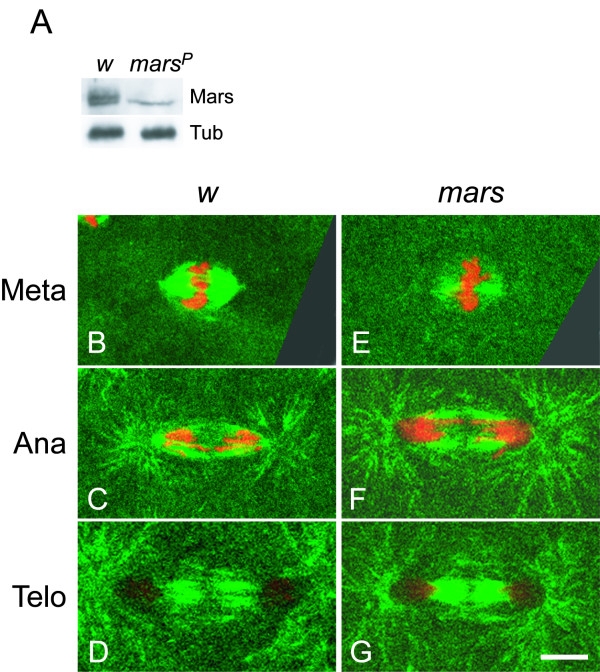
**Embryos with reduced *mars *activity exhibit shorter and less dense mitotic spindles**. (A) Immunoblotting of Mars protein extracted from 30 syncytial blastoderm embryos at metaphase with an anti-Mars antibody. A 135-kDa protein was detected and is labeled as Mars on the right. α-Tub was served as the loading control. (B-G) Embryos at 28.5°C were immunostained with anti-α-Tub (green) and anti-γ-Tub (green) and anti-phospho-histone H3 (red) antibodies. The morphology of the mitotic spindles at metaphase, anaphase and telophase in *w*^*1118 *^(B-D) and *mars *(E-G) embryos are shown. The scale bar is 5 μm

Yang and Fan have shown that two bands are detected in extracts from *Drosophila *S2 cells by immunoblotting with anti-Mars antibody and that the upper band consists of Mars that is phosphorylated [38]. To reveal whether both forms of Mars protein are differentially reduced in *mars *embryos, Mars protein in metaphase embryos was determined by immunoblotting. Using extracts from *Drosophila *embryos, reproducibly, two bands located close together and with equal intensity were able to be detected in this study (Fig. 4A); this differs from other studies, where a single band has been found [30,43]. In *mars *embryos, the amount of the unphosphorylated Mars, the lower band, was less than half of that in *w*^*1118 *^embryos and the phosphorylated Mars was barely detectable (lane *mars*^*P *^in Fig. 4A). These results plus the association of phosphorylated Mars with taxol-stabilized microtubules [38] indicated that the phosphorylated Mars, but not the unphosphorylated protein, is associated with mitotic spindles. The drastic reduction in phosphorylated Mars on the mitotic spindles might cause gynandromorphs to appear in progeny from parents homozygous for *mars*^*P*^.

Mitotic spindles in *mars *embryos from 1 to 2.5 hours at 28.5°C were examined using immunostaining with anti-α-Tub, anti-γ-Tub and anti-phospho-histone H3 antibodies. In metaphase *mars *embryos, the length and the density of mitotic spindles (7.50 ± 0.17 μm and 28.53 ± 3.31 au) were significantly shorter and less, respectively, than those in *w*^*1118 *^embryos (11.98 ± 0.16 μm and 53.71 ± 7.03 au) (compare Fig. 4B with 4E; *p *< 0.001 and 0.05, respectively). At anaphase and at telophase, neither the length nor the density of mitotic spindles was substantially affected in the *mars *embryos (Figs 4C, D, F and 4G). These results indicated that the polymerization of the mitotic spindles in *mars *embryos is delayed, but not entirely prevented.

### Asynchronous chromosome segregation is observed in mars embryos

Consistent with the fact that polymerization of mitotic spindles is affected in *mars *embryos, progression of mitotic events in patches of nuclei, each of which contained at least two nuclei, was obviously delayed comparing to those in adjacent nuclei (Figs 2C and 2D) in 13% of *mars *embryos (n = 32). A possible explanation for these results is that the mitotic spindles lacking phosphorylated Mars pull the chromosomes toward the spindle poles inefficiently, which results in both asynchronous chromosome segregation (Fig. 2D) and embryos pausing at anaphase (Table 2).

**Table 2:** Mitotic index of embryos with reduced *mars *activity or *tlk *overexpression

		Mitosis phases					
			
Genotype of parents	Interphase	Pro	Meta	Ana	Telo	Mixed *	Number of embryos
*w*^*1118*^	59.3%	10.9%	23.2%	3.1%	2.0%	1.5%	543
*mars*^*P*^	49.9%	17.4%	17.1%	11.2%	0.4%	4.0%	546
*GCN4*>*tlk*^#^	39.2%	15.1%	25.0%	5.3%	12.6%	2.8%	602

Embryos were immunostained with anti-histone H3 antibody and scored under a fluorescent microscope.

*: Embryos have at least two different mitotic phases. For examples, nuclei at the anterior of the embryo are at telophase whereas those at the posterior remain at metaphase.

#: *GCN4*>*tlk *are from females carrying both *UASP*-*tlk *and *GCN4-GAL4 *transgenes mated with *UAST*-*tlk *males.

### Genetic interaction of *mars* with *tlk* is required for accurate chromosome segregation

Without a proper genetic marker, identification of gynandromorphs is difficult if the X/O cells do not locate to the posterior end of adult females. Therefore, *mars*^*P *^males that also carry *yellow *(*y*) and *singed *(*sn*) markers on the X chromosome were used to measure the gynandromorphy rate. Interestingly, the frequencies of non-disjunction, namely the ratios of y^- ^sn^- ^males to total males, in the male progeny from *w*^*1118*^; *mars*^*P *^females crossed with *y*^*1*^*w*^*1*^*sn*^*3*^; *mars*^*P *^at 24°C and 28.5°C were 0.6% and 1.1%, respectively (Table 3), which are much higher than the gynandromorphy rate in the *mars *parent. Hereafter, we measured the non-disjunction rate instead of gynandromorphy because it was a simpler procedure.

**Table 3:** The non-disjunction resulting from *mars *loss-of-function is enhanced by reduced *tlk *activity

Genotype of mothers	Number of male progeny	y^- ^w^- ^sn^- ^male progeny
*w*^*1118*^	979	0 (0.0%)
*w*^*1118*^; *mars*^*P*^	476 374*	3 (0.6%) 4 (1.1%)
*w*^*67c23 *^*tlk*^*27-9*^/*w*^*1118*^	579	0 (0.0%)
*w*^*67c23 *^*tlk*^*Δ14*^/*w*^*1118*^	909 1071*	1 (0.1%) 4 (0.4%)
*w*^*67c23 *^*tlk*^*27-9*^/*w*^*1118*^;*mars*^*P*^/*+*	997 636*	1 (0.1%) 1 (0.2%)
*w*^*67c23 *^*tlk*^*Δ14*^/*w*^*1118*^;*mars*^*P*^/*+*	838 1170*	0 (0.0%) 3 (0.3%)
*w*^*67c23 *^*tlk*^*27-9*^/*w*^*1118*^;*mars*^*P*^	398 411*	5 (1.2%) 25 (6.1%^#^)
*w*^*1118 *^*tlk*^*Δ14*^/*w*^*1118*^;*mars*^*P*^	926 1188*	5 (0.5%) 52 (4.4%^#^)

Ten to twelve 2-day old females with the genotypes indicated in the left column were crossed with *y*^*1*^*w*^*1*^*sn*^*3*^; *mars*^*P *^males. Each cross was set up in at least five vials and transferred into new vials after three days. After the second transfer, the parents were discarded. The flies were incubated at 24°C or 28.5°C ± 0.5°C. The latter is indicated by an asterisk. Male progenies were counted for five consecutive days. The percentage of y^- ^w^- ^sn^- ^offspring over total males is shown in parenthesis. Number sign, #, represents a significant difference between *mars*^*P *^and *tlk*^*27-9 or Δ14*^/*+*;*mars*^*P *^at 28.5°C as determined by the Student's *t*-test (*p *< 0.01).

To test whether *mars *interacts with *tlk *genetically, dosage-dependent interaction experiments were performed with two *tlk *alleles, Δ14 and 27-9. The hatching rate of embryos from *tlk*^*27-9*^/+; *mars*^*P *^females was 48%, a further reduction from the 67% for *mars *embryos (Table 1), indicating that *mars *genetically interacts with *tlk*.

The frequencies of non-disjunction in male progenies from *tlk*^*27-9*^/+; *mars*^*P *^and *tlk*^*Δ14*^/+; *mars*^*P *^females at 28.5°C were 6.1% and 4.4%; these rates were much higher than those from *mars*^*P *^(1.1%) or *tlk*^*Δ14*^/+ females (0.4%) (Table 3). This increase in frequency of non-disjunction was also temperature dependent, since there was no substantial increase in progeny from *tlk*^*27-9*^/+; *mars*^*P *^and *tlk*^*Δ14*^/+; *mars*^*P *^females (1.2% and 0.5%, respectively) at 24°C when compared to *mars*^*P *^or *tlk*^*Δ14*^/+ females. These results indicated that *mars *genetically interacts with *tlk *and that the interaction is involved in ensuring accurate chromosome segregation.

### *mars* acts in parallel to *tlk* during chromosome segregation

To reveal whether mitosis is severely affected in embryos with reductions in both *mars *and *tlk *activity, embryos from *tlk*^*Δ14*^/+; *mars*^*P *^females crossed to *mars*^*P *^males (abbreviated as *tlk*/*mars *embryos) at 28.5°C; these embryos were then immunostained using anti-α-Tub, anti-γ-Tub and anti-phospho-histone H3 antibodies. A series of images along the z axis were stacked to observe the distribution of chromosomes and mitotic spindles. In embryos from females heterozygous for *tlk*^*Δ14*^, the chromosomes before entering anaphase were aligned at the spindle midzone as indicated by a bracket in Fig. 2B and the morphology of the mitotic spindles appeared normal (*tlk*^*Δ14*^/+; compare Fig. 2A with 4B). In almost all *tlk*/*mars *embryos at either metaphase or anaphase (n = 70), however, the asynchrony was so severe that it was impossible to distinguish what phase an embryo belonged to. In addition to the asynchrony that was observed in *mars *embryos (Fig. 2D), asynchronous chromosome segregation was observed in embryos where most of the nuclei were likely at anaphase (Fig. 2E). Despite the severe asynchrony during chromosome congression or segregation, the morphology of the mitotic spindles in *tlk*/*mars *embryos was not significantly different from that in *mars *embryos (compare Fig. 2E with 2C). These results suggested that *mars *acts in parallel to *tlk*.

**Figure 5:**
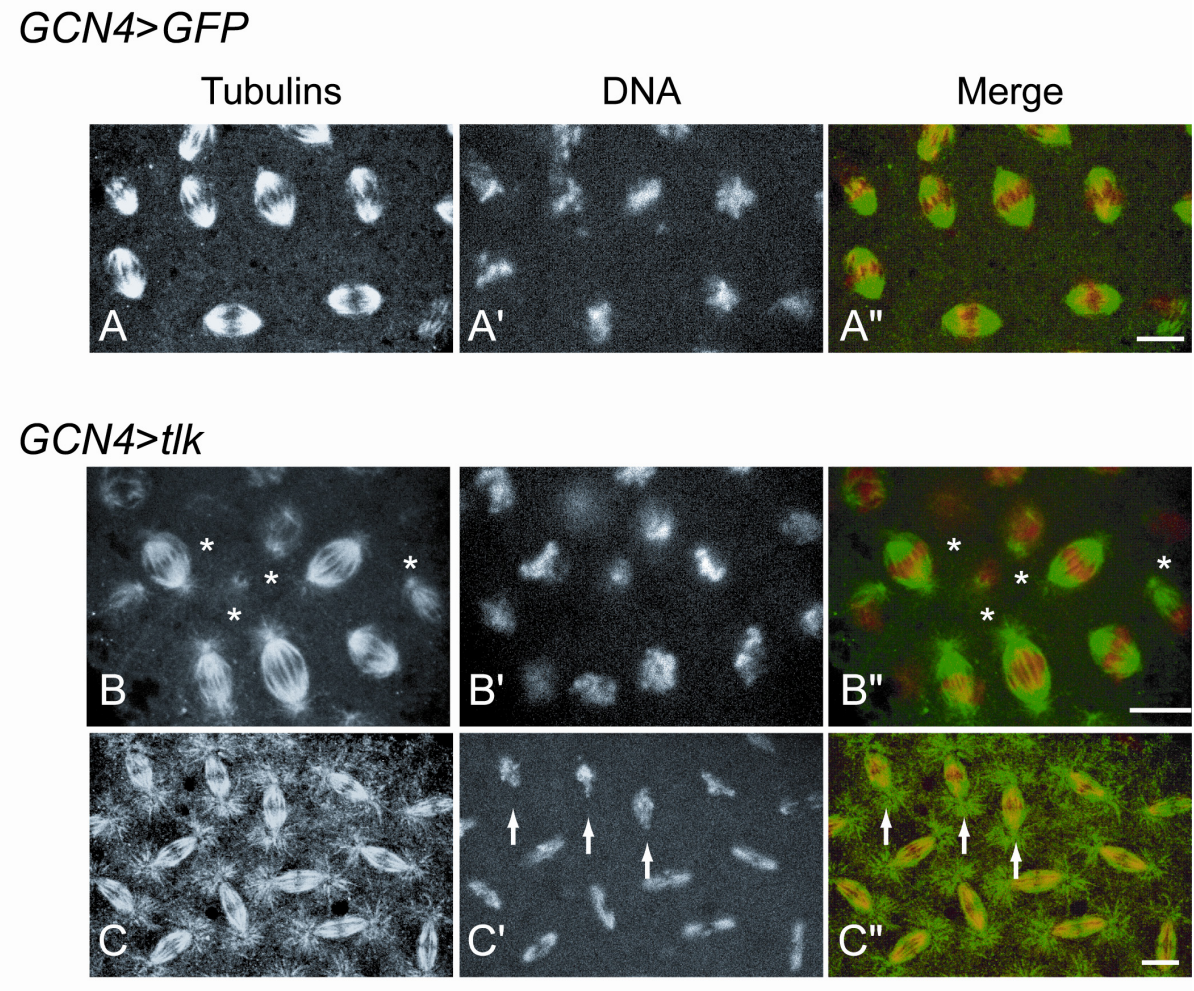
**Mitotic defects in embryos with *tlk *overexpression are similar to those observed in *mars *embryos**. Embryos with either *GFP *(*GCN4*>*GFP*) or *tlk *overexpression (*GCN4*>*tlk*) at 28.5°C were immunostained with anti-α-Tub (green) and anti-γ-Tub (green) antibodies. The chromosomes were stained by Hoechst 33342 (red). (A) *GFP *overexpression in metaphase embryo serves as a negative control. (B) Most of the mitotic spindles were normal in metaphase embryos when *tlk *was overexpressed. A few nuclei showed corrupted mitotic spindles that also appear to have less chromosomal DNA and these are indicated by asterisks. (C) A patch of nuclei with less condensed chromosomes at anaphase is indicated by arrows and the chromosomes seem to remain unseparated. These two phenotypes were also observed in *GCN4*>*tlk *embryos at 24°C. The scale bars are 10 μm.

**Figure 6:**
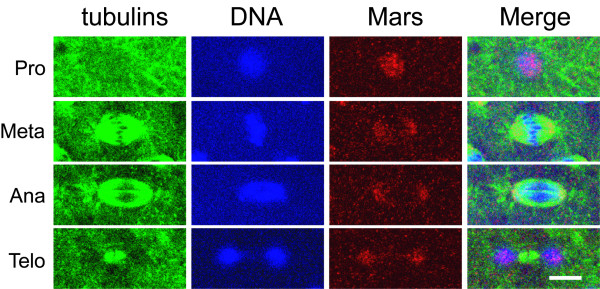
**Localization of Mars on mitotic spindles is unaffected by *tlk *overexpression**. Embryos at 28.5°C from *GCN4*>*tlk *females crossed with *UAST*-*tlk *males were immunostained with anti-Mars (red), anti-α-Tub (green) and anti-γ-Tub (green) antibodies. The chromosomes were stained with Hoechst 33342 (blue). The Mars patterns in *GCN4*>*tlk *embryos at prophase, metaphase, anaphase and telophase are similar to those in *w*^*1118 *^embryos (Fig. 1). The scale bar is 5 μm.

To explore the parallel nature of the interaction between *mars *and *tlk *further, we tested whether the morphology of the mitotic spindles and Mars localization was affected in embryos overexpressing *tlk*. *GCN4*>*tlk *embryos at 24°C were immunostained with anti-Mars, anti-α-Tub and anti-γ-Tub antibodies. Images were processed as described above. Overexpression of *tlk *induced a delayed progression of mitotic events, which was manifest as several observable features similar to those seen in *mars *embryos. Firstly, the fraction of embryos at prophase was similar (Table 2). Secondly, patches of nuclei with delayed chromosome congression were seen in 30% of the metaphase embryos (n = 150) (Fig. 5B). Thirdly, at least one patch of nuclei exhibited delayed chromosome segregation with chromosome bridges in half of anaphase embryos (n = 32) (Fig. 5C). Despite the similarity of these effects to those observed in *mars *embryos, neither the length nor the density of most mitotic spindles at metaphase was substantially affected by *tlk *overexpression (compare Figs. 5A with 5B). In agreement with this, *tlk *overexpression did not either affect the localization of Mars protein to mitotic spindles (Fig. 6) or decrease the quantity of acetylated tubulin (data not shown) that exists in the stable microtubules [2]. Taken together with the different subcellular localizations of Mars and Tlk, which localize to spindle microtubules and chromosomes respectively (this study; [17,30,43]), these results supported the notion that Mars functions in parallel to Tlk.

### Both mars and tlk activities are required for cells to correctly progress through chromosome segregation

**Figure 7:**
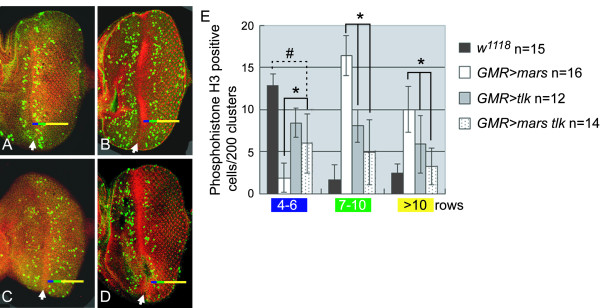
**The M-phase arrest induced by *mars *overexpression is suppressed by *tlk *overexpression**. Eye-antennal discs were dissected from the third-instar larvae and immunostained with anti-phospho-histone H3 antibody (green) and phalloidin (red) to label the M-phase cells and the neuronal clusters, respectively. The genotypes for panel A to D are *w*^*1118 *^(A), *GMR*>*mars *(B), *GMR*>*tlk *(C)and *GMR*>*mars tlk *(D). The anterior of the discs is arranged toward the left. The arrows indicate the morphogenetic furrows (MF). The region behind the MF is divided into three distinct domains, namely I (4–6), II (7–10) and III (>11 rows of neuronal clusters), as described by Baker and Yu (2001) [45]. The color codes for the three domains are blue, green and red for domains I, II and III, respectively. (E) From each disc, the M-phase cells in two hundred clusters (10 clusters parallel and 20 clusters perpendicular to MF) were measured and plotted as a histogram. The representation and number of discs measured for each column are shown on the right. The statistical significance of M-phase cells presenting in the three regions was determined using the Student's *t*-test. The numbers of M-phase cells in the eye discs with overexpression of both *mars *and *tlk *differ significantly from those overexpressing *mars *or *tlk *alone and are also different from the *w*^*1118 *^discs. These differences are indicated by asterisks and #, respectively (*p *< 0.05).

Our previous results have shown that *mars *overexpression induces metaphase arrest in eye discs, with chromosomes attached to spindle monotelicaly in some cases [29]. Based on the role of Tlk-1 acting as a cofactor of Aur-B [3,19,23], we next asked the question whether *tlk *overexpression could overcome the metaphase arrest. To test this, we counted M-phase cells in the three different domains behind morphogenetic furrow (MF) as classified by Baker and Yu (2001) [45]. Reproducibly, *mars *overexpression resulted in cells in domain I being retained at interphase and in many cells in domains II and III being retained at M-phase (Figs. 7B and 7E); this should be compared with the fact that most M-phase cells appear in domain I of *w*^*1118 *^discs (Fig. 7A and 7E). Similarly, *tlk *overexpression induced a delayed progression of mitosis, but to a lesser extent (Figs. 7C and 7E). When both genes were co-overexpressed, the number of M-phase cells in domain III was reduced to a level close to that of the wild-type, showing that the metaphase arrest induced by *mars *overexpression was suppressed by *tlk *overexpression (Fig. 7E). These results indicated that a balance between *mars *and *tlk *activities is required for cells to progress through mitosis correctly.

## Discussion

In this study, we have examined the genetic interaction between *mars *and *tlk *in terms of chromosome fidelity. Progeny from *mars *parents showed a low percentage of gynandromorphs and non-disjunction. The decreased fidelity is a result of delayed polymerization of mitotic spindles as shown by shorter and thinner mitotic spindles in embryos with reduced *mars *activity, which is consistent with the recent findings reported by Tan et al [30]. In addition, the non-disjunction rate was significantly increased by removing one copy of *tlk *at the non-permissive temperature.

Tlk is a substrate and cofactor of Aur-B, which is a chromosome passenger protein and localizes to centromeres during the prophase to metaphase-to-anaphase transition [3,19,23]. Aur-B destabilizes kinetochore-bound microtubules locally to promote syn-to-amphitelic attachment [24,46,47]. Microtubule dynamics are important for the assembly of mitotic spindles, as well as for the segregation of chromosomes captured by the mitotic spindles at kinetochores [48,49]. Our results showed that *tlk *overexpression suppresses the metaphase arrest induced by *mars *overexpression. This metaphase arrest is a result of abnormal polymerization of mitotic spindles, resulting in the syntelic or monotelic attachment in some cases [29]. The fact that Tlk-1 mediates the activation of Aur-B kinase [3] provides an explanation. It is likely that Aur-B kinase activity is elevated by *tlk *overexpression, which destabilizes kinetochore-bound microtubules more rapidly; this counteracts the polymerization of mitotic spindles induced by *mars *overexpression. This will result in the suppression of the metaphase arrest. The local rapid-turnover of microtubules induced by the increased Aur-B activity may also cause deterioration in the spindle microtubules bound to kinetochores, which could lead to asynchrony without an obvious effect on the mitotic spindles in embryos overexpressing *tlk*. Therefore, Mars and Tlk function to promote polymerization of the mitotic spindle and to elevate Aur-B kinase activity, respectively. Both are important for the syntelic-to-amphitelic transition, this hypothesis being supported by the increase in non-disjunction and the observation of more severe asynchrony when both gene activities were reduced. Nevertheless, determination of the epistatic relationship between *aur-B *and *tlk *awaits suitable *aur-B *mutants.

Our results showed that a *mars *hypomorph exhibits subtle defects in chromosome fidelity and viability, which are temperature dependent. Little or no phenotype has been reported for loss-of-function mutants in many comprehensive studies, such as the *hhoA *gene in bacteria [50], the *PPH21*, *MDM17 *and *ISS1 *genes in yeast [51-53] and the *Suppressor of fused *gene in fly [54]. These phenotypes then become more obvious when the organism is incubated at an elevated temperature and/or when one copy of an interacting locus is removed. The products of these genes are either components or redundant factors in protein complexes. For example, deletion of the *ISS1 *gene has no significant effect on yeast growth; however, when combined with *sec24*, which codes for a component in the v-SNARE complex, the combination of *iss1 *and *sec24 *in yeast becomes lethal. Furthermore, *iss1 *overexpression is able to suppress the mutation in *sec24*, indicating that Iss1 can replace Sec24 [51]. In a similar way, the enhancement of the mild mitotic defects in flies caused by reduced *mars *activity when the incubation temperature is elevated or when one copy of *tlk *is removed suggests that both Mars and Tlk proteins participate together in one or more protein complexes that are required for chromosome segregation.

## Conclusion

This study showed that there was a low but significant rate of gynandromorphy and non-disjunction in progeny from parents homozygous for *mars*, which indicates that Mars is required for accurate chromosome segregation. The decreased fidelity was due to an inability to polymerize mitotic spindles correctly and this led to chromosome aberrations in embryos with reduced *mars *activity. Chromosome fidelity was significantly decreased to a further extent when one copy of *tlk *was removed in addition to the reduction in *mars *activity. The results from cytological studies indicated that *mars *acts in parallel to *tlk *and that a balance between *mars *and *tlk *activity is required for cells to progress through mitosis correctly and ensure chromosome fidelity.

## Competing interests

The authors declare that they have no competing interests.

## Authors' contributions

HHL carried out most of the experiments and provided valuable inputs into the manuscript. HYH observed the nuclear cleavage in embryos with reduced *mars *activity by the time-lapse recording and found the inaccurate chromosome segregation, which led to the finding on gynandromorphy and non-disjunction. Both CSC and GJL participated in design of the study and writing the manuscript. GJL also performed part of the experiments. All authors have read and approved the final manuscript.
